# Supplementary material for: Driving innovation: When design collaboration becomes open source, how do reward mechanisms and in-process feedback act as catalysts
Source: PLoS One. 2025 Jul 2;20(7):e0327482. doi: 10.1371/journal.pone.0327482 (PMC12221004; doi:10.1371/journal.pone.0327482)
Supplement: S2 Data — (DOCX) [file pone.0327482.s002.docx]

**Follow-up Questionnaire on Experimental Experience**

**I. Personality Traits**

**Question 1** In Experiment 1 (Round Fan - Scores Not Disclosed), how many design proposals did you generally prefer to generate in each round? [Single-choice question]

| **Option** | **Subtotal** | **Percentage** |
| --- | --- | --- |
| 1 | 4 | 20% 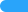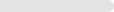 |
| 2-3 | 8 | 40% 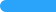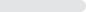 |
| 4-6 | 6 | 30% 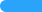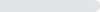 |
| More than 6 | 2 | 10% 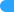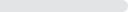 |

**Question 2** In Experiment 2 (Folding Fan - Scores Disclosed), how many design proposals did you generally prefer to generate in each round? [Single-choice question]

| **Option** | **Subtotal** | **Percentage** |
| --- | --- | --- |
| 1 | 3 | 15% 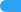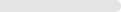 |
| 2-3 | 16 | 80% 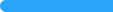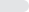 |
| 4-6 | 1 | 5% 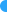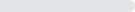 |
| More than 6 | 0 | 0% 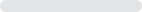 |

**Question 3** In Experiment 1 (Round Fan - Scores Not Disclosed), please rank the factors influencing the number of proposals you generated in each round: [Ranking question]

| **Option** | **Comprehensive Score** | **1st Place** | **2nd Place** | **3rd Place** | **4th Place** | **5th Place** | **6th Place** | **7th Place** | **Subtotal** |
| --- | --- | --- | --- | --- | --- | --- | --- | --- | --- |
| Personal Time | 5.8 | 11(61.11%) | 4(22.22%) | 3(16.67%) | 0(0%) | 0(0%) | 0(0%) | 0(0%) | 18 |
| Quantity of Design Inspirations and Ideas | 4.95 | 5(29.41%) | 7(41.18%) | 4(23.53%) | 0(0%) | 0(0%) | 1(5.88%) | 0(0%) | 17 |
| Personal Interest in the Task | 4.15 | 3(18.75%) | 5(31.25%) | 2(12.5%) | 4(25%) | 2(12.5%) | 0(0%) | 0(0%) | 16 |
| Others' Proposal Quantities | 3.2 | 1(6.67%) | 2(13.33%) | 5(33.33%) | 3(20%) | 0(0%) | 4(26.67%) | 0(0%) | 15 |
| Competition Rules and Reward Incentives | 2.2 | 0(0%) | 0(0%) | 1(7.14%) | 5(35.71%) | 3(21.43%) | 5(35.71%) | 0(0%) | 14 |
| Competition Stage (Round) | 1.9 | 0(0%) | 0(0%) | 0(0%) | 2(15.38%) | 8(61.54%) | 3(23.08%) | 0(0%) | 13 |
| Others | 0.05 | 0(0%) | 0(0%) | 0(0%) | 0(0%) | 0(0%) | 0(0%) | 1(100%) | 1 |

**Question 4** In Experiment 2 (Folding Fan - Scores Disclosed), please rank the factors influencing the number of proposals you generated in each round: [Ranking question]

| **Option** | **Comprehensive Score** | **1st Place** | **2nd Place** | **3rd Place** | **4th Place** | **5th Place** | **6th Place** | **7th Place** | **8th Place** | **Subtotal** |
| --- | --- | --- | --- | --- | --- | --- | --- | --- | --- | --- |
| Personal Time | 6.45 | 9(50%) | 5(27.78%) | 3(16.67%) | 0(0%) | 1(5.56%) | 0(0%) | 0(0%) | 0(0%) | 18 |
| Quantity of Design Inspirations and Ideas | 5.65 | 7(41.18%) | 4(23.53%) | 2(11.76%) | 3(17.65%) | 0(0%) | 0(0%) | 1(5.88%) | 0(0%) | 17 |
| Personal Interest in the Task | 4.5 | 2(12.5%) | 5(31.25%) | 2(12.5%) | 2(12.5%) | 2(12.5%) | 3(18.75%) | 0(0%) | 0(0%) | 16 |
| Scoring Situation | 4.15 | 1(6.25%) | 3(18.75%) | 3(18.75%) | 4(25%) | 2(12.5%) | 2(12.5%) | 1(6.25%) | 0(0%) | 16 |
| Competition Rules and Reward Incentives | 3.05 | 0(0%) | 1(5.88%) | 3(17.65%) | 0(0%) | 3(17.65%) | 4(23.53%) | 6(35.29%) | 0(0%) | 17 |
| Competition Stage (Round) | 3.05 | 1(6.67%) | 0(0%) | 1(6.67%) | 3(20%) | 5(33.33%) | 2(13.33%) | 3(20%) | 0(0%) | 15 |
| Others' Proposal Quantities | 3 | 0(0%) | 1(7.14%) | 4(28.57%) | 2(14.29%) | 1(7.14%) | 3(21.43%) | 3(21.43%) | 0(0%) | 14 |
| Others | 0.1 | 0(0%) | 0(0%) | 0(0%) | 0(0%) | 0(0%) | 0(0%) | 0(0%) | 2(100%) | 2 |

**Question 5** In Experiment 1 (Round Fan - Scores Not Disclosed), did you have a preference for the number of proposals you cited? [Single-choice question]

| **Option** | **Subtotal** | **Percentage** |
| --- | --- | --- |
| Never cite | 5 | 25% 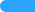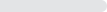 |
| Occasionally cite 1 per round | 10 | 50% 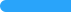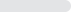 |
| Almost always cite 1 per round | 3 | 15% 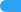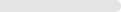 |
| Occasionally cite 2-3 per round | 2 | 10% 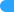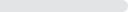 |
| Almost always cite 2-3 or more per round | 0 | 0% 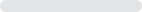 |

**Question 6** In Experiment 2 (Folding Fan - Scores Disclosed), did you have a preference for the number of proposals you cited? [Single-choice question]

| **Option** | **Subtotal** | **Percentage** |
| --- | --- | --- |
| Never cite | 7 | 35% 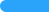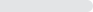 |
| Occasionally cite 1 per round | 9 | 45% 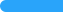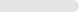 |
| Almost always cite 1 per round | 1 | 5% 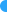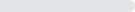 |
| Occasionally cite 2-3 per round | 3 | 15% 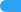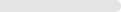 |
| Almost always cite 2-3 or more per round | 0 | 0% 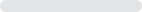 |

**Question 7** In Experiment 2, when selecting reference proposals, did you care more about the proposal's score or your personal preference? [Single-choice question]

| **Option** | **Subtotal** | **Percentage** |
| --- | --- | --- |
| Don't care about either | 1 | 5% 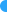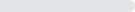 |
| Care more about scores | 9 | 45% 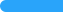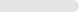 |
| Care more about personal judgment | 6 | 30% 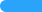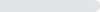 |
| Care about both equally | 4 | 20% 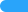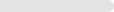 |

**Question 8** In Experiment 1 (Round Fan - Scores Not Disclosed), did you prefer to cite your own proposals or others' proposals? [Single-choice question]

| **Option** | **Subtotal** | **Percentage** |
| --- | --- | --- |
| Prefer to cite my own | 7 | 35% 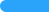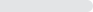 |
| Prefer to cite others' | 1 | 5% 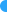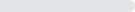 |
| Like both | 4 | 20% 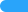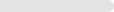 |
| Unsure, depends on the situation | 6 | 30% 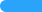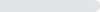 |
| Dislike both | 2 | 10% 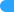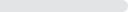 |

**Question 9** In Experiment 2 (Folding Fan - Scores Disclosed), did you prefer to cite your own proposals or others' proposals? [Single-choice question]

| **Option** | **Subtotal** | **Percentage** |
| --- | --- | --- |
| Prefer to cite my own | 5 | 25% 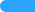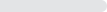 |
| Prefer to cite others' | 5 | 25% 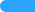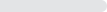 |
| Like both | 4 | 20% 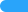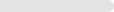 |
| Unsure, depends on the situation | 5 | 25% 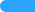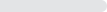 |
| Dislike both | 1 | 5% 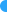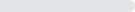 |

**Question 10** Throughout the entire experiment, did you pay attention to others' citation situations, such as who someone cited? [Single-choice question]

| **Option** | **Subtotal** | **Percentage** |
| --- | --- | --- |
| Completely not attentive | 3 | 15% 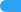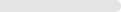 |
| Not very attentive | 6 | 30% 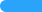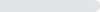 |
| Moderate | 4 | 20% 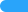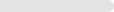 |
| Relatively attentive | 7 | 35% 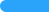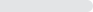 |
| Very attentive | 0 | 0% 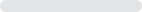 |

**Question 11** Throughout the entire experiment, would you tend to cite a proposal if you saw others citing it? [Single-choice question]

| **Option** | **Subtotal** | **Percentage** |
| --- | --- | --- |
| Completely not | 6 | 30% 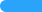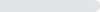 |
| Not very likely | 9 | 45% 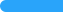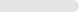 |
| Moderate | 2 | 10% 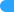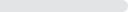 |
| Relatively likely | 3 | 15% 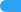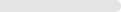 |
| Definitely | 0 | 0% 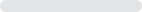 |

**Question 12** In Experiment 1 (Round Fan - Scores Not Disclosed), how did you feel about the design sense of your own proposals? [Single-choice question]

| **Option** | **Subtotal** | **Percentage** |
| --- | --- | --- |
| Very dissatisfied | 1 | 5% 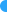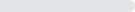 |
| Somewhat dissatisfied | 1 | 5% 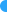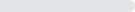 |
| Neutral | 4 | 20% 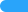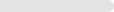 |
| Relatively satisfied | 12 | 60% 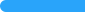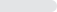 |
| Very satisfied | 2 | 10% 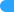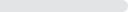 |

**Question 13** During Experiment 1, before the results were announced, how confident were you that your designed proposals would win an award? [Single-choice question]

| **Option** | **Subtotal** | **Percentage** |
| --- | --- | --- |
| Completely unconfident | 0 | 0% 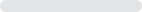 |
| Somewhat unconfident | 6 | 30% 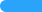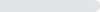 |
| Neutral | 9 | 45% 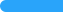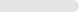 |
| Relatively confident | 4 | 20% 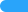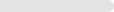 |
| Very confident | 1 | 5% 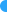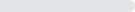 |

**Question 14** In Experiment 2 (Folding Fan - Scores Disclosed), how did you feel about the design sense of your own proposals? [Single-choice question]

| **Option** | **Subtotal** | **Percentage** |
| --- | --- | --- |
| Very dissatisfied | 0 | 0% 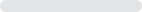 |
| Somewhat dissatisfied | 1 | 5% 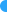 |
| Neutral | 5 | 25% |
| Relatively satisfied | 14 | 70% |
| Very satisfied | 0 | 0% |

**Question 15** During Experiment 2, before the scores were fed back and the results were announced in each round, how confident were you that your designed proposals would win an award? [Single-choice question]

| **Option** | **Subtotal** | **Percentage** |
| --- | --- | --- |
| Completely unconfident | 0 | 0% |
| Somewhat unconfident | 7 | 35% |
| Neutral | 7 | 35% |
| Relatively confident | 5 | 25% |
| Very confident | 1 | 5% |

**Question 16** Would you worry that others borrowing your proposals would reduce your chances of winning? [Single-choice question]

| **Option** | **Subtotal** | **Percentage** |
| --- | --- | --- |
| Completely not worried | 6 | 30% |
| Not very worried | 9 | 45% |
| Moderate/Unsure | 3 | 15% |
| Somewhat worried | 2 | 10% |
| Very worried | 0 | 0% |

**Question 17** Do you consider yourself a designer with many creative ideas and rich imagination? [Single-choice question]

| **Option** | **Subtotal** | **Percentage** |
| --- | --- | --- |
| No creative ideas at all | 0 | 0% |
| Relatively few creative ideas | 1 | 5% |
| Moderate | 15 | 75% |
| Relatively many creative ideas | 3 | 15% |
| Extremely many creative ideas | 1 | 5% |

**Question 18** For most tasks (not too simple nor extremely difficult), do you prefer to collaborate with other designers or design independently? [Single-choice question]

| **Option** | **Subtotal** | **Percentage** |
| --- | --- | --- |
| Prefer independent design | 9 | 45% |
| Prefer 2-3 person collaboration | 8 | 40% |
| Prefer 3-5 person collaboration | 1 | 5% |
| Prefer 5+ person team collaboration | 2 | 10% |

**Question 19** Please rate your tendency towards each of the following, where 0 means no tendency in that aspect: [Matrix slider]

Characteristics of extroverted designers: Cheerful, emotionally excitable, with drastic mood changes and impulsive tendencies, but quick to calm down; rapid reactions, straightforward and enthusiastic; Rich imagination, many ideas, not stuck in convention, fond of innovation and being different. Thoughts easily diverge, hard to focus, etc.

Characteristics of introverted designers: Stable mood, not easily impulsive; rigorous and meticulous, with thorough and comprehensive consideration of things, having a strong sense of overall situation, able to conduct in-depth and thorough research and thinking. Slower thinking, relatively rigid, with many rules, less variation in work forms.

| **Row Title** | **Average Value** |
| --- | --- |
| Extroverted Tendency | 2.4 |
| Introverted Tendency | 2.6 |
|  | Subtotal: 5 Average: 2.5 |

**II. Experience and Satisfaction**

**Question 20** Regarding the reward rules implemented by your group, how fair do you think they were? [Single-choice question]

| **Option** | **Subtotal** | **Percentage** |
| --- | --- | --- |
| Very unfair | 0 | 0% |
| Somewhat unfair | 0 | 0% |
| Neutral | 1 | 5% |
| Relatively fair | 16 | 80% |
| Very fair | 3 | 15% |

**Question 21** Regarding the reward rules implemented by your group, how attractive do you think they were? (Attractiveness refers to the extent to which the reward model motivates your interest in participating in similar activities in the future and your willingness to obtain the reward) [Single-choice question]

| **Option** | **Subtotal** | **Percentage** |
| --- | --- | --- |
| Completely unattractive | 0 | 0% |
| Somewhat unattractive | 2 | 10% |
| Neutral | 8 | 40% |
| Relatively attractive | 10 | 50% |
| Very attractive | 0 | 0% |

**Question 22** In Experiment 1, where all proposals were publicly displayed in each round but scores were not disclosed, and judges gave a final result at the end, how satisfied were you with this evaluation method? [Single-choice question]

| **Option** | **Subtotal** | **Percentage** |
| --- | --- | --- |
| Very dissatisfied | 0 | 0% |
| Somewhat dissatisfied | 5 | 25% |
| Neutral | 9 | 45% |
| Relatively satisfied | 6 | 30% |
| Very satisfied | 0 | 0% |

**Question 23** In Experiment 2, where proposals were publicly displayed and evaluation scores were announced in each round, how satisfied were you with this evaluation method? [Single-choice question]

| **Option** | **Subtotal** | **Percentage** |
| --- | --- | --- |
| Very dissatisfied | 0 | 0% |
| Somewhat dissatisfied | 2 | 10% |
| Neutral | 3 | 15% |
| Relatively satisfied | 12 | 60% |
| Very satisfied | 3 | 15% |

**Question 24** Impact of public evaluation: [Matrix single-choice question]

| **Question\Option** | **Strongly Disagree** | **Disagree** | **Neutral** | **Agree** | **Strongly Agree** |
| --- | --- | --- | --- | --- | --- |
| Made me feel pressured or urgent | 2(10%) | 1(5%) | 11(55%) | 5(25%) | 1(5%) |
| Getting high scores/score improvement gave me a sense of achievement | 0(0%) | 0(0%) | 2(10%) | 11(55%) | 7(35%) |
| Increased my enthusiasm for designing | 0(0%) | 1(5%) | 6(30%) | 11(55%) | 2(10%) |
| Made me feel more involved | 0(0%) | 1(5%) | 5(25%) | 12(60%) | 2(10%) |
| Increased my desire to win awards | 1(5%) | 1(5%) | 3(15%) | 13(65%) | 2(10%) |
| Not disclosing evaluations made me feel confused and unsure where to start | 2(10%) | 3(15%) | 5(25%) | 7(35%) | 3(15%) |
| Not disclosing evaluations made me feel nervous | 3(15%) | 5(25%) | 9(45%) | 3(15%) | 0(0%) |

**Question 25** In traditional design competition models, participants generally compete individually, without public sharing of all competitors' proposals as in this experiment. What do you think? [Matrix single-choice question]

| **Question\Option** | **Strongly Disagree** | **Disagree** | **Neutral** | **Agree** | **Strongly Agree** |
| --- | --- | --- | --- | --- | --- |
| Traditional competition model with no public disclosure of proposals | 0(0%) | 5(25%) | 12(60%) | 3(15%) | 0(0%) |
| A competition model where participants publicly disclose their own proposals and can view others' proposals (imitation/plagiarism not allowed, meaning you must create completely different proposals) | 0(0%) | 2(10%) | 9(45%) | 8(40%) | 1(5%) |
| A competition model where participants publicly disclose their own proposals, can view and are allowed to cite/borrow others' proposals (borrowing is legalized; if a borrowed work wins an award, the original creator receives certain rewards) | 0(0%) | 1(5%) | 7(35%) | 11(55%) | 1(5%) |

**Question 26** The rule of citing/borrowing others' proposals and providing attribution requires designers' integrity. Between pursuing self-interest and achieving mutual benefit, what do you think? [Single-choice question]

| **Option** | **Subtotal** | **Percentage** |
| --- | --- | --- |
| Absolutely self-interested; no one will follow the rules | 0 | 0% |
| Predominantly self-interested; most people will not follow the rules | 6 | 30% |
| Neutral | 6 | 30% |
| Predominantly mutually beneficial; most people will follow the rules | 8 | 40% |
| Absolutely mutually beneficial; everyone will follow the rules | 0 | 0% |
