# Supplementary material for: Driving innovation: When design collaboration becomes open source, how do reward mechanisms and in-process feedback act as catalysts
Source: PLoS One. 2025 Jul 2;20(7):e0327482. doi: 10.1371/journal.pone.0327482 (PMC12221004; doi:10.1371/journal.pone.0327482)
Supplement: Attachment — Submitted filename: Response to Reviewers.doc [file pone.0327482.s004.doc]

Responses to Reviewers’ Comments for Manuscript PONE-D-25-04408

**Driving innovation: When design collaboration becomes open source,**

**how do reward mechanisms and in-process feedback act as catalysts**

Addressed Comments for Publication to PLoS ONE

By

Boqun Xu, Kexin Xu, Yuning Zhu, Yiting Zhou, Xiaojian Liu

**Dear Editors,**

We thank all the editors and reviewers for the valuable comments and suggestions. We have carefully revised the manuscript to enhance its clarity and facilitate the understanding of the readers. Our point-to-point responses are presented in the following. We hope that the revision would satisfactorily address the comments and concerns of the editors and reviewers.

Sincerely yours,

Yuning Zhu

Department of Design and Architecture
Zhejiang University of Technology
288 Liuhe Road, Xihu District, Hangzhou, Zhejiang Province, China
E-mail: [zhuyuning@zjut.edu.cn](mailto:zhuyuning@zjut.edu.cn)

**Responses:**

To Editor........................................................................................................ p3-p7

To Reviewer #1.............................................................................................. p8-p10

To Reviewer #2.............................................................................................. p11-p13

**Note:** To enhance the legibility of this response letter, all the editor’s and reviewers’ comments are typeset in boxes. Some of the modified parts are marked in blue font. For more modified parts, please refer to the revised manuscript. The respective parts in the manuscript are highlighted to indicate changes.

# Response to Editor

**Summary Comment 1**

The conceptualization of “referencing behavior” as a mediating variable requires deeper theoretical integration. Reviewer 1 rightly notes that the current classification (self- vs. other-reference) remains descriptive. Anchoring this construct in established motivational frameworks (e.g., attribution theory, social identity theory) would enhance explanatory power and generalizability.

**Response S1:** Thank you for your summary.

We appreciate that this thoughtful concern aligns with the query raised by Reviewer 1. The corresponding revisions have been thoroughly addressed in ***Response 1.1***.

**Summary Comment 2**

Similarly, the causal pathways connecting motivation, behavior, and outcomes need elaboration. Integrating questionnaire data to model psychological mechanisms (e.g., intrinsic motivation, social expectations) could bridge this gap.

**Response S2:** Thank you for the comment.

We recognize that psychological mechanisms do play an important role in this study. According to this question, we have systematically examined mechanisms related to rewards and competition, and supplemented theories such as intrinsic motivation, risk preference, and social comparison tendencies. For instance, the interviewees' scores of fairness and attractiveness might reflect an individual's risk preference. The interviewees' attitudes towards feedback intervention indicated the adjustment of their internal driving forces to their personal creation or citation behaviors, ultimately leading to differences in the outcome of the work and the collaborative relationship. The specific answer can be seen in ***Response 1.3***.

**Summary Comment 3**

The small, context-specific sample (N=20) limits the external validity of findings. While the experimental design controls for internal validity, the paper should explicitly address how institutional or demographic homogeneity might constrain broader applicability. Expanding the “Limitations” section to discuss these factors is essential.

**Response S3:** Thank you for your pertinent advice.

We sincerely appreciate your careful attention to these aspects, which directly reflect comment 1.2. We have explicitly outlined the potential limitations of our research findings and corresponding precautions (see ***lines 757-765 on page 34***), and the specific modifications are as follows in the blue part:

As the number of participants in collaborative communities grows, behaviors become more unpredictable, potentially leading to disruptive processes driven by competition [95], including referencing behaviors. Our experiment was conducted under the precondition of participant integrity. However, the lack of large-scale crowd collaboration and the relatively concentrated sample limit the generalizability of our findings to broader crowdsourcing contexts. Results may vary across different professional backgrounds, design tasks (e.g., color matching vs. modeling), or offline settings. We acknowledge the broader utility of large samples in research and recommend attention to the value of small-sample studies for generating reproducible and novel insights [1]. As an exploratory study providing a conceptual framework, this work encourages future research to involve more diverse and larger participant groups to improve data validity and result reliability.

[1] Li X, Liu J, Gao W, Cohen GL. Challenging the N-Heuristic: Effect size, not sample size, predicts the replicability of psychological science. PLoS ONE. 2024 Aug 23;19(8): e0306911.

**Summary Comment 4**

As noted by Reviewer 2, providing descriptive statistics on participant characteristics (e.g., prior collaboration experience, domain expertise) and testing for baseline differences between experimental groups would bolster confidence in the results’ integrity.

**Response S4:** Thank you for the comment.

Based on the suggestion of Reviewer 2, the descriptive data of the experimental personnel have been supplemented accordingly ***(see Table 1)***. Potential variables have been controlled as much as possible during the recruitment of subjects and the grouping of personnel to minimize the baseline differences to the greatest extent.

**Summary Comment 5**

The current focus on citation path length overlooks richer network dynamics (e.g., centrality, cohesion). Incorporating metrics from social network analysis could better explain how reference structures mediate creative collaboration.

**Response S5:** Thank you for your reminder.

We believe this is consistent with the question raised by Reviewer 1. The specific answer can be seen in ***Response 1.4***. Overall, we have conducted additional data analysis, and the new results are now incorporated in the revised manuscript to provide further support and enhance the robustness of our findings.

**Summary Comment 6**

The study could leverage questionnaire responses more systematically to unpack psychological drivers behind observed behaviors, thereby enriching the causal narrative.

**Response S6:** Thank you for the comment.

As the questions raised in summary comment 2, we carefully examined the relationship between the questionnaire and psychological driving factors in the revised draft, the answer and specific modifications here can be referred to ***Response 1.3***.

**Summary Comment 7**

The “Discussion” section should articulate clearer managerial implications. For instance, how might platform designers balance reward structures with feedback mechanisms to optimize creativity? How do referencing behaviors inform policies for knowledge attribution in open collaborations? Addressing these questions would enhance relevance for applied audiences.

**Response S7:** Thank you for your specific explanation.

We review and find that the original practical significance indeed did not provide sufficient suggestions for the application audience. We summarize the research conclusions and provide referenceable action suggestions for project initiators, platform managers and designers respectively, to help them improve efficiency or achieve better results in actual implementation as much as possible. ***The modified contents are as follows***:

Several scholars have argued that open source sustainability is a latent key issue [15,92]. This study offers insights for managing open-source design collaboration, communities can benefit in multiple ways. First, project outcomes are influenced by various factors, and rules should be carefully designed based on the core objectives of the crowdsourcing project. For the project initiators, if the goal is to generate a large volume of ideas to inspire creativity, a quota allocation with high rewards can boost competition and increase output, though it may not support long-term sustainability. If the goal is to secure a few high-quality designs for direct use or expand the scope of collaboration as much as possible, a contribution-based allocation encourages collaboration and the development of superior designs, which also promotes continued participation.

Second, for the platform managers, involving evaluators (project owners) in the collaboration process and providing timely feedback is recommended. This facilitates faster convergence of designs, enhances innovation, and enriches designers' experiences, thereby increasing their willingness to participate in future projects. However, caution is needed regarding the simultaneous implementation of the CA pattern and in-process feedback. For open-source design crowdsourcing projects, it is advisable to establish a system integrating reference tags and traceability chains to protect designers' original knowledge and make referencing behaviors an explicit identifier of participants' professional identities. This helps resolve intellectual property disputes and strengthens collaboration networks by linking resource access to contribution.

Finally, for platform designers, project selection should align with personal goals and skills. Those aiming to accumulate work quantity and maximize rewards may prioritize projects with QA pattern, while setting appropriate expectations for lower returns. Those seeking reputation and creative growth may be more suitable for the CA pattern projects without feedback, which fully integrate collaborative networks and foster frequent user interactions—similar to the GitHub model. ***(page 32, lines 714-737)***

**Summary Comment 8**

The paper’s academic contribution could be sharpened by situating findings within broader debates on motivation in crowdsourced innovation or knowledge-sharing economies.

**Response S8:** Thank you for your valuable suggestions on the research contribution.

Your question is of summary significance, guiding us to summarize the relevant analyses of the psychological mechanisms and motivation theories in this revision. The detailed analysis can be found in , and we have also included a summary discussion in the ***Practical Implications section***, the details are as follows. We sincerely thank you again for your effort.

Group communication and interaction can generate collective intelligence [93], further enhancing participants' understanding of tasks and improving the quality of creative output [94]. The experimental results also revealed how differences in participants’ motivations affect knowledge contribution, output quality, and sustainability, which are the core issues in crowdsourced innovation. We suggest strengthening the stimulation of intrinsic motivation by autonomy, competence and a sense of relationship in an appropriate way. According to Social Exchange Theory, individuals weigh costs and benefits in social interactions; poorly designed incentives may lead to free-riding or monopolistic behavior, which poses risks to knowledge-sharing economies. We hope to identify effective collaboration models and foster the healthy expansion of collaborative networks. Through such feedback and knowledge sharing, both designers' skills and the overall quality of project community improve. This study offers valuable guidance for both participants and project owners, providing practical guidance for effectively implementing open-source collaboration in product design. ***(page 33, lines 740-748)***

# Response to Reviewer #1

**Comment 1.1**

The theoretical construction of key variables appears somewhat empirical.

"Referencing behavior," as a central mediating variable, is primarily categorized based on observational distinctions (self-reference vs. other-reference), without being grounded in a broader or transferable motivational framework, The current treatment leans toward descriptive classification rather than explanatory modeling, Stronger alignment with motivation theories-such as attribution theory or social identity theory-would enhance the conceptual robustness.

**Response 1.1:** Thank you for your helpful suggestions.

The original exposition was too simple and subjective. Reference is indeed related to attribution and personal identification. In the revised version, we described in detail the introduction basis and significance of the concept of "reference behavior" at the beginning of ***Participants' behavior and attitude***. For details, please refer to ***lines 395-403 on page 18***.

**Comment 1.2**

Limitations in sample size and generalizability.

The study involves a relatively small sample (N=20) drawn from a single institutional or organizational context. This raises concerns about the generalizability of the findings across broader populations, platforms, or collaborative scenarios. This limitation should be more explicitly addressed in the limitations section.

**Response 1.2:** Thank you for the pertinent comment.

We originally briefly mentioned the limitations but not in detail. Due to the limitation of the sample size, it is impossible to calculate the effect size to improve the reliability. In the revised manuscript, we clearly stated the possible directions of limitation and precautions of the research results (***Page 34. lines 757-765***).

**Comment 1.3**

Causal mechanisms linking motivation, behavior, and outcomes are underdeveloped.

While the experimental results capture the behavioral effects of reward and feedback, the underlying psychological mechanisms-such as risk aversion, social expectations, or intrinsic drive-remain insufficiently explored, A more systematic integration of the questionnaire results could help model these mediating pathways more rigorously.

**Response 1.3:** Thank you for your question and recommendation.

We acknowledge the important role of psychological factors in shaping participants' behaviors and outcomes. Following your suggestions, we have systematically investigated the psychological mechanisms related to rewards and competition, and supplemented theories such as intrinsic motivation, risk preference, and social comparison tendencies. We optimized the conclusions based on the original questionnaire data and further linked the potential role of psychological mechanisms on behavior and outcomes. (All the yellow sections in the revised manuscript, ***pages 25-27, lines 554-606***).

Initially, we did not emphasize this aspect due to the extensive existing research on psychological factors in reward and feedback. Our primary focus was on the role of variables in behavior and results, and relatively weakens the mediating role of statistical methods in observing psychological mechanisms. However, as originally mentioned in the overview section of ***Reward Mechanisms (page 6)***, intrinsic motivation and external rewards can affect outcome quality (e.g., achievement, fairness expectations). And in-process feedback may stimulate competitive psychology and bring about positive or negative effects ***(page 8)***. Still, questionnaires and interviews were conducted and interviews were conducted during the experiment. We believe that such data are equally significant for the research.

**Comment 1.4**

The analysis of reference network structure lacks methodological depth.

The current description of the "reference chain" primarily focuses on the length of citation paths without incorporating network-analytic metrics such as node centrality, diffusion dynamics, or structural cohesion. This limits the explanatory power of the reference network in mediating creative output and collaborative engagement.

**Response 1.4:** Thank you for your valuable suggestions.

We think this is an excellent suggestion. We have conducted additional data analysis, and the new results are now incorporated in the revised manuscript to provide further support and enhance the robustness of our findings. To make it clear, we have added the above data and discussions in the main text (All the yellow sections in the revised manuscript from ***pages 18-23, lines 406 to 514***).

In addition, after adding new analysis, we adjusted the sequence of the paragraph structure to optimize the logic. We organized the new network analysis and the original citation chain length analysis into ***Collaborative network and reference features (page 18)***, and separately adjusted the original award result analysis to ***Awards of design work (page 23)***.

We have also made certain supplements in the ***summary section*** (All the yellow sections in the revised manuscript on ***pages 28-29, lines 617 to 642***). Hopefully, you would be satisfied with these supplements.

**Comment 1.5**

The discussion and managerial implications could be further developed.

While the paper presents interesting findings, the practical takeaways for platform designers, innovation managers, or policy makers are somewhat limited. Strengthening this section would enhance the study's relevance to both academic and applied audiences.

**Response 1.5:** Thank you for your helpful suggestions.

We review and find that the original practical significance indeed did not provide sufficient suggestions for the application audience. We summarize the research conclusions and provide referenceable action suggestions for project initiators, platform managers and designers respectively, to help them improve efficiency or achieve better results in actual implementation as much as possible (All the yellow sections in the revised manuscript from ***pages 32-33, lines 714 to 737***).

# Response to Reviewer #2

**Comment 2.1**

First of all, can you explain the strengths of the methodology of this study, why it is suitable for this study and can you clearly answer the research questions of the article.

**Response 2.1:** Thank you for bringing these concerns to our attention.

As you suggested, it is important to emphasize these insights in the paper. Below is our explanation.

As noted in ***the summary of the literature review (page 9)***, open-source collaboration is relatively novel and significant in design area yet under-researched, we aim to explore how certain variables influence key outcomes, in order to facilitate the development of this collaborative model. **The core research question is: How do reward mechanisms and in-process feedback affect innovation outcomes (both quantity and quality) and collaborative behaviors (such as referencing patterns and participation willingness) in open-source design collaboration.** Specifically, we want to investigate the following questions:

1.How different reward mechanisms impact design output, and through what forms of individual motivation this influence occurs;

2.Whether in-process feedback acts as a form of informational guidance that enhances or inhibits innovation and collaboration efficiency;

3. How reward mechanisms and in-process feedback influence the structure of creative iteration networks.

By addressing these issues, the study aims to provide actionable insights into optimizing “reward structure + feedback strategy” on collaborative platforms, offering practical guidance for balancing competition and cooperation in open-source design.

In terms of methodology, we employed **an empirical experimental approach** aligned with the research goals. While the sample size is limited, this method remains a foundational tool in social science research and is especially useful for early-stage exploratory studies. The experiment featured **multi-round iterative collaboration in an open-task format** to capture dynamic processes and rich data. We used a **2×2 factorial design** to systematically isolate the effects of each variable on collaborative innovation, thereby enhancing the experiment’s rigor and controllability. Additionally, we utilized **a mixed-methods analysis combining quantitative and qualitative data**. This allowed us to capture the direct causal relationships between variables and behavior at the data level, while also revealing underlying psychological mechanisms through surveys and interviews. This multi-dimensional approach enabled comprehensive measurement of key indicators and interpretation of participant motivation, supporting the study’s layered research design.

Overall, these methods combined can closely simulate real-world settings like referencing, disclosure, and feedback, while minimizing external noise. The structure makes the process quantifiably collectible, facilitating the replication and expansion of follow-up research.

We clearly defined the research questions and experimental methods in the revised manuscript (***page 9, lines 209-217***).

**Comment 2.2**

The second is, can the paper show the descriptive characteristics of the experimental subjects or the differences between the two groups, especially the characteristics that may affect the results of the experiment, thus increasing the credibility of the results.

**Response 2.2:** Thank you for your valuable suggestions.

We apologize for the confusion. The descriptive data of the experimental personnel have been supplemented accordingly ***(page 11, Table 1)***. Potential variables have been controlled as much as possible during the recruitment of subjects and the grouping of personnel to minimize the baseline differences to the greatest extent. We believe this helps to enhance the credibility of the results.

**Table 1. Participants' descriptive characteristics**

| **Group** | **Type** | **Personnel characteristics** | | | | | | | | | |
| --- | --- | --- | --- | --- | --- | --- | --- | --- | --- | --- | --- |
| Group 1  (QAM) | Code | A | B | C | D | E | F | G | H | I | J |
|  | Gender | M | F | M | M | M | M | F | F | F | F |
|  | Years of Engagement in Design | 18 | 9 | 3 | 7 | 7 | 7 | 3 | 4 | 7 | 6 |
| Group 2  (CAM) | Code | a | b | c | d | e | f | g | h | i | j |
|  | Gender | M | M | M | M | M | M | F | F | F | F |
|  | Years of Engagement in Design | 19 | 6 | 3 | 7 | 7 | 7 | 3 | 4 | 7 | 6 |
